# The Impact of Vaccination against SARS-CoV-2 on Health Outcomes and Hospital Visits after Omicron Infection in Children and Adolescents Aged 5–18 Years: A Danish Nation-Wide Cohort Study

**DOI:** 10.3390/vaccines11121766

**Published:** 2023-11-27

**Authors:** Selina Kikkenborg Berg, Helle Wallach-Kildemoes, Line Ryberg Rasmussen, Ulrikka Nygaard, Henning Bundgaard, Annette Kjær Ersbøll, Louise Bering, Lau Caspar Thygesen, Susanne Dam Nielsen, Anne Vinggaard Christensen

**Affiliations:** 1 Department of Cardiology, Rigshospitalet, Copenhagen University Hospital, Inge Lehmanns Vej 7, 2100 Copenhagen, Denmarklouise.bering.pedersen@regionh.dk (L.B.);; 2 Faculty of Health and Medical Sciences, University of Copenhagen, Blegdamsvej 3B, 2200 Copenhagen, Denmark; 3 Department of Paediatrics and Adolescents Medicine, Rigshospitalet, Copenhagen University Hospital, Blegdamsvej 9, 2100 Copenhagen, Denmark; 4 National Institute of Public Health, University of Southern Denmark, Studiestræde 6, 1455 Copenhagen, Denmark; 5 Department of Infectious Disease, Rigshospitalet, Copenhagen University Hospital, Blegdamsvej 9, 2100 Copenhagen, Denmark

**Keywords:** COVID-19, vaccine, BNT162b2, children and adolescents, Omicron

## Abstract

This study investigates the impact of vaccination against SARS-CoV-2 on health outcomes and hospital contacts in children and adolescents aged 5–18 years infected with the SARS-CoV-2 Omicron variant, comparing previously vaccinated with unvaccinated. Using national register data, vaccinated and unvaccinated Danish children and adolescents with a positive SARS-CoV-2 test between 1 January and 31 March 2022 (Omicron dominance period) were included. The Prior Event Rate Ratio (PERR) was used to explore differences in hospital contacts (hospitalizations and emergency room (ER) visits), while Inverse Treatment Probability Weighted (IPW) risk ratios were used to explore the risk of severe health outcomes within six weeks following SARS-CoV-2 infection. Vaccinated 5–11-year-old girls had fewer visits to the ER compared to unvaccinated ones, PERR 0.92 (95% CI 0.84–1.00). Vaccinated 5–11-year-old boys had fewer hospitalizations (PERR 0.79 (0.64–0.99)) and more ER visits (PERR 1.13 (1.04–1.22)) compared to unvaccinated ones. An unadjusted and significant lower risk of febrile seizure among vaccinated 5–11-year-olds compared to unvaccinated ones was found (risk ratio 0.12 (0.04–0.39), *p* ≤ 0.01. No significant differences were found for severe conditions or for croup or pneumonia in either age group. The results indicate a modest protective effect of the vaccine in terms of hospital contacts, but no protective effect on health outcomes after SARS-CoV-2 Omicron infection in this population of Danish children and adolescents.

## 1. Introduction

At present, the COVID-19 mRNA vaccine BNT162b2 (Pfizer-BioNTech) is authorized for emergency use in children and adolescents aged 5–18 years by the U.S. Food and Drug Administration (FDA) and the European Medicines Agency (EMA). The vaccine was approved for emergency use among 12–16-year-olds in the spring and summer of 2021 and for 5–11-year-olds in November 2021. The vaccine has shown efficacy against laboratory-confirmed SARS-CoV-2 (Alpha (B.1.1.7) and Beta (B.1.351) variants) in more than 90% in children and adolescents aged 5–11, 12–15, and 16–17 years [1,2,3]. At the time when vaccination against SARS-CoV-2 was initiated, the B.1.617.2 (Delta) variant was dominant. For this variant of SARS-CoV-2, the BNT162b2 vaccine proved to be effective in protecting against hospitalization and death following infection in American and Puerto Rican populations of adolescents above 12 years of age. These studies covered periods from December 2020 to September/October 2021 [4,5]. In a systematic review including several COVID-19 vaccines, the efficacy of protection against infection with SARS-CoV-2 decreased by 20–30% over time in populations above 12 years of age. The effectiveness of the vaccines against severe disease was stable above 70% after 6 months [6].

In November 2021, the B.1.1.529 (Omicron) variant of SARS-CoV-2 emerged and quickly became dominant. This new variant seemed to cause fewer symptoms [7], but at the same time it was highly infectious and studies indicated that the vaccines were not as effective against the Omicron variant [8].

In a systematic review and meta-analysis including 14 studies focusing on COVID-19 vaccination and infection with the Omicron variant in children and adolescents, the pooled vaccine effectiveness (VE) against symptomatic COVID-19 was 45% in children and 73% in adolescents [9].

In an American case–control study, the estimated VE for 5–11-year-olds and 12–15-year-olds was 60.1% and 59.5% 2–4 weeks after vaccination, respectively. After two months, the estimated VE was 28.9% and 16.6% in the two age groups [10]. Another American case–control study including 5–18 year-olds evaluated the protection of the vaccine against hospitalization after COVID-19 infection for the Delta and Omicron variant. In the 12–18-year-olds, the VE against COVID-19-related hospitalization was higher during the Delta period than during the Omicron period (92% vs. 40%) 2–22 weeks after vaccination. Among children aged 5–11 years, the VE was 68% against COVID-19-related hospitalization in the Omicron period [11].

Thus, previous research indicates that the VE of the BNT162b2 vaccine is lower for the Omicron variant than the Delta one in terms of protection against infection, severe disease, and hospitalization. Further, it indicated that the VE declines rapidly with time since infection and that the effects of the vaccine may differ between children and adolescents.

The objective of the present study was to investigate the impact of vaccination against SARS-CoV-2 on health outcomes and hospital contacts in children and adolescents aged 5–18 years infected with the SARS-CoV-2 Omicron variant comparing previously vaccinated with unvaccinated subjects.

## 2. Methods

### 2.1. Setting

Denmark began free-of-charge, mass vaccination against SARS-CoV-2 for those aged 16–19, 12–15, and 5–11 years in 2021 on 15 May, 14 July, and 28 November, respectively. Public, free-of-charge SARS-CoV-2 reverse-transcriptase polymerase chain reaction (PCR) testing was available from late May 2020 until March 2023, with Denmark being one of the countries testing the largest proportion of its population [12]. At the individual level, the results from SARS-CoV-2 tests were recorded in the Danish Microbiology Database (MiBa) [13].

During the pandemic, restrictions on healthcare contacts and school attendance were implemented in Denmark to varying degrees following periods with high SARS-CoV-2 transmission. The Omicron variant became dominant in Denmark by 1 January 2022 [14].

All Danish children were sent home from school from 15 December 2021 until 5 January 2022. After that, all children from 1st grade and up, and staff were encouraged to undergo screening tests twice a week.

### 2.2. Study Design and Population

In this real-life, nation-wide cohort study, we used data from the following Danish registries: The Danish Civil Registration System [15], the Danish Vaccination Register (DDV) [16], the MiBa [13], the Danish National Patient Register (DNPaR) [17], and the Danish National Prescription Registry (DNPrR) [18]. Data were linked at the individual level, applying an encrypted version of the unique Danish Civil Registration Number issued to all Danish citizens at birth or at the date of work and/or residence permit.

We aimed to investigate outcomes up to six weeks after Omicron infection among children and adolescents (5–18 years old) who were fully vaccinated prior to infection and those who were not (unvaccinated). Hence, the study population included Danish children and adolescents with a positive SARS-CoV-2 test between 1 January and 31 March 2022 (Omicron dominance period). Children were regarded as fully vaccinated 14 days after the second vaccine dose.

Excluded were individuals who tested SARS-CoV-2 positive before the inclusion period, individuals who tested SARS-CoV-2 positive within 14 days after the second vaccine dose, individuals vaccinated with COVID-19 vaccines other than Pfizer-BioNTech (BNT162b2), individuals who had only received the first dose of the vaccine by the end of the inclusion period, unvaccinated individuals who turned 5 years old in December 2021, individuals vaccinated prior to the age-schedule, and individuals who were unvaccinated at the time of infection but vaccinated during the six weeks follow-up period (in order not to mix up with potential vaccine adverse effects).

The study was permitted by the Danish Data Protection Agency (P-2021-195) and registered at clinicaltrials.gov (NCT04786353). Ethics committee approvals are not required for register-based studies in Denmark. Register data access was granted by The Danish Health Data Authority (FSEID 00005625).

### 2.3. Index-Dates and Age

Two index-dates were applied in the analyses with Indexdate-Vac (index-date) corresponding to the first possible vaccination date in accordance with the age-specific vaccination rollout, and Indexdate-Inf corresponding to the date of the first SARS-CoV-2 positive test. To enable assignment of an index-date among the unvaccinated in a similar period of the Danish COVID-19 epidemic as the vaccinated, we opted for simplicity to apply the age-specific first date of possible first dose vaccination as the index-date (15 May 2021, for 16–18-year-olds, 14 July 2021, for 12–15-year-olds and 28 November 2021, for 5–11-year-olds). To avoid mixing up with potential adverse outcomes following vaccination, the index-date was applied for specific outcomes prior to infection compared with outcomes after positive SARS-CoV-2 test applying Indexdate-Inf.

While age among the vaccinated corresponds to the age (in years) at the date of first vaccine dose (index-date), age (in years) among the unvaccinated was assigned based on their vaccinated peers and their infection-age (assigned to the Wednesday in the week when they tested SARS-CoV-2 positive). For each infection-age and week of positive SARS-CoV-2 tests, sub-groups of vaccinated and unvaccinated were created. The unvaccinated were assigned an age corresponding to either the infection-age or the infection-age minus one (depending on birthday late or early in the calendar year). We assigned the same proportion of the youngest among the unvaccinated an age equal to infection-age as the proportion among their vaccinated peers with an age when vaccinated equal to infection-age. Conversely, the same proportion of the oldest were assigned an age equal to infection-age minus one. Throughout the analyses, we applied the age at (possible) vaccination, dividing age into two age-groups (5–11 years and 12–18 years).

### 2.4. Variables

#### 2.4.1. Exposure and Follow-Up Period

Individuals were included for six weeks follow-up after being tested SARS-CoV-2 positive as exposed (vaccinated) or unexposed (unvaccinated), according to the below criteria:

Vaccinated: individuals with a positive SARS-CoV-2 test at least 14 days after the second vaccine dose of Pfizer-BioNTech vaccine against SARS-CoV-2 (BNT162b2).

Unvaccinated: individuals unvaccinated when tested SARS-CoV-2 positive and still unvaccinated up to 6 weeks after the test, i.e., during the follow-up period.

#### 2.4.2. Outcomes

We explored two outcomes for subjects after being tested SARS-CoV-2 positive:

- Hospital visits:
  Hospital admissions and emergency room (ER) visits. ER visits were defined as acute contacts lasting less than 12 h, otherwise they were defined as hospitalizations. Planned outpatient visits were excluded. Outcomes were measured as counts of visits.
- SARS-CoV-2 infection health implications (Supplementary Table S1):
  - Registered ICD-10 diagnoses corresponding to known severe health outcomes following SARS-CoV-2 infection (Delta variant and earlier).
    Included list of first ever diagnoses: Multisystem Inflammatory Syndrome in Children (MIS-C), myocarditis, venous thromboembolism, Guillain–Barré syndrome, and encephalitis. We constructed a binary variable, termed severe conditions; this took a value of yes if at least one of the listed diagnoses were observed during follow-up.
  - Other less severe registered ICD-10 diagnoses, accounting for possible registration of the diagnoses four years prior to index-date: pneumonia, croup, and Febrile seizure.

### 2.5. Descriptive Table: Baseline Characteristics

To describe the differences in baseline characteristics (i.e., prior to Indexdate-Inf) among the vaccinated and unvaccinated, information on age, sex, medical history (ICD-10 codes, see Supplementary Table S2) prior to positive SARS-CoV-2 test, and current medicine use (ATC-codes, see Supplementary Table S2) six months prior to Indexdate-Inf were included.

While medical history on somatic conditions was based on discharge diagnoses for in- and outpatients including both primary and secondary diagnoses, medical history on psychiatric conditions was restricted to in-patient discharge diagnoses and primary diagnoses.

### 2.6. Statistical Analyses

(1) Hospital visits as outcome measure.

The analytical approach was inspired by Kildegaard et al., 2000 [19]. The Prior Event Rate Ratio (PERR) methodology was applied for exploring the impact of being vaccinated or not on hospital admissions and emergency room visits within 6 weeks after infection. The PERR approach assumes that the ratio post (Indexdate-Inf) to prior period (1 year prior to index-date) of ‘vaccinated to unvaccinated outcome event rate’ reflects the effect of the post-period ‘vaccinated to unvaccinated outcome event rate’ adjusted for measured and unmeasured time-independent confounders [20]. For each outcome measure of interest, PERR estimates were calculated through simple division of outcome rates, stratified by age-group (5–11 and 12–18 years). We used bootstrapping [21] with 200 replicates randomly sampled with replacement to obtain 200 PERR estimates, calculating the 95% confidence intervals (CI) for the original PERR estimate through the percentile distribution of the bootstrapped PERR estimates. For PERR estimates >1, the pre- versus post-difference in outcome event rate among the vaccinated was higher than among the unvaccinated. The analyses of healthcare visits were stratified by sex and age group.

(2) Adverse health events as outcome measure.

Risk ratio was applied to explore the impact of being vaccinated or unvaccinated on health outcomes listed in Supplementary Table S1 within the six weeks after SARS-CoV-2 infection.

In line with Kildegaard et al. [19], we estimated the propensity score weighted risk ratio with a 95% CI [22] using a generalized linear model for binary outcomes (Stata: binomial regression with identity resp. log link = ‘binreg’), comparing outcomes in the vaccinated with the unvaccinated. We estimated the propensity score of being vaccinated by means of the Inverse Treatment Probability Weighting (IPW) [23], including in the model age, sex, comorbidities (medical history), and current medicine use (i.e., prior to index-date).

For the five included severe conditions following SARS-CoV-2 infection, we applied the composite outcome binary variable severe conditions, excluding individuals with prior registration of one of the included outcomes. We thereby included only individuals with first-ever severe diagnoses. Due to very few events, the analyses were unstratified.

For each of the less severe registered ICD-10 diagnoses (pneumonia, croup, and febrile seizure), we accounted for possible registration of the specific diagnosis within four years prior to index-date by including a prior-diagnosis variable (yes/no) in the propensity score model. The analyses were stratified by age-group. Any recorded febrile seizure in adolescents was considered a coding error as febrile seizures occur in children aged six months up to about 5 years [24]. Hence, a risk estimate regarding febrile seizure could not be calculated for the 12–18-year-olds.

Data management and statistical analyses were performed using StataCorp. 2021. Stata Statistical Software: Release 17.

## 3. Results

### 3.1. Participants

Table 1 shows the baseline characteristics of the included children and adolescents prior to infection with Omicron by age-groups. The population consisted of 103,712 unvaccinated and 64,063 vaccinated children aged 5–11 years and 29,538 unvaccinated and 223,325 vaccinated adolescents aged 12–18 years. While the proportion of girls among the 5–11-year-olds was similar between the vaccinated and unvaccinated (48.3% versus 48.9%), the proportion for the 12–18-year-olds was higher in the vaccinated than in the unvaccinated (50.1% versus 48.2%). Among the 5–11-year-olds, the mean age at possible vaccination was 7.5 years (standard deviation (SD) 2.0) and 8.8 years (SD 1.9) for the unvaccinated and vaccinated, respectively. For the 12–18-year-olds, the mean age at possible vaccination was 13.8 years (SD 1.7) for the unvaccinated and 14.6 years (SD 1.8) for the vaccinated. As per design, the mean ages at Omicron infection were higher than mean vaccination ages at possible vaccination. This was most pronounced among the 12–18-year-olds with a longer timespan between the vaccination roll-out during summer 2021 and infection during the first three months of 2022, Table 1.

The prevalence of comorbidities was similar between the vaccinated and unvaccinated. However, the prevalence of asthma was higher in vaccinated 5–11-year-olds and current/recent use of inhaled short-acting-beta2-agonist was higher among vaccinated 12–18-year-olds compared to the unvaccinated. Furthermore, current/recent use of antibiotics was slightly higher in the vaccinated group in 12–18-year-olds compared to the unvaccinated, Table 1.

### 3.2. Hospital Visits

Vaccinated girls aged 5–11 years had a lower rate-ratio of visits to the ER compared to the unvaccinated, PERR 0.92 (95% CI 0.84–1.00). Compared to the unvaccinated, vaccinated 5–11-year-old boys had a lower rate-ratio of hospitalization (PERR 0.79 (95% CI 0.64–0.99)) and a higher rate-ratio of visits to the ER (PERR 1.13 (95% CI 1.04–1.22)). Vaccinated and unvaccinated 12–18-year-olds had similar risk of hospitalization and visits to the ER, Table 2.

### 3.3. Severe Health Events

No significant risk-estimates were found in the combined measure on severe conditions, including MIS-C, myocarditis, venous thromboembolism, Guillain–Barré syndrome, and encephalitis, Table 3.

Within six weeks after SARS-CoV-2 infection, an unadjusted and significant lower risk of febrile seizure among vaccinated 5–11-year-olds compared to unvaccinated ones was found (risk ratio 0.12 (0.04–0.39), *p* ≤ 0.01), and by an IPW adjusted risk-ratio estimate of 0.30 (0.09–1.08), *p* = 0.07. When comparing the vaccinated and unvaccinated, no significant risk-estimates were seen as to developing croup or pneumonia. However, there was a tendency towards a lower risk of pneumonia among 12–18-year-olds, reflected by an IPW adjusted risk-ratio estimate of 0.44 (0.15–1.27), *p* = 0.13, Table 4.

## 4. Discussion

Six weeks after SARS-CoV-2 infection, the 5–11-year-old vaccinated girls had a lower rate of visits to the ER compared to unvaccinated girls. Conversely, 5–11-year-old vaccinated boys had a higher rate of visits to the ER and a lower rate of hospitalizations compared to unvaccinated boys. For health outcomes, no significant differences were found in vaccinated 5–11-year-olds or 12–18-year-olds compared to unvaccinated children.

### 4.1. Strengths and Limitations

A main strength of this study is the use of real-life data from the nationwide Danish administrative registers. The DDV [16] and the MiBa [13] contain complete and updated data on COVID-19 vaccination and SARS-CoV-2 infection, and with Denmark being one of the countries testing the largest proportion of the population [12], this strengthens the validity of our results. From the DNPaR [17], we have complete individual-level information on hospital contacts and ICD-10 codes given at hospital contacts. Thus, we were able to follow the study population six weeks after SARS-CoV-2 infection.

In line with a previous Danish study [19] the PERR methodology was applied to adjust for any time-independent confounders. Thus, any differences in healthcare use between the vaccinated and unvaccinated cannot be explained by differences in habitual healthcare use.

Comprehensive characteristics of the populations of vaccinated and unvaccinated children and adolescents are presented to allow for comparison between groups.

External validity is supported by Denmark adhering to international guidelines for vaccination of adolescents.

Despite applying a binary variable for the five rare health outcomes included, counts of several of the included health outcomes were low. This could be expected when exploring rare health conditions in children and adolescents, and lead to concerns about power. However, confidence intervals for the estimates are not too wide.

### 4.2. Interpretation

Among vaccinated girls aged 5 to 11 years, fewer visits to the ER were found in the present study (PERR 0.92 (0.84–1.00)). An American study found that the VE of two vaccine doses against COVID-19-associated emergency departments or urgent care encounters was 51% (30–65%) among children aged 5–11 years during Omicron dominance, thus also indicating a protective effect of the vaccine. For children aged 12–15 years, VE was 45% (30–57%), and for those aged 16–17 years, it was 34% (8–53%) [25].

A lower rate of hospitalization was found among vaccinated boys aged 5–11 years old (PERR 0.79 (0.64–0.99)). In an American population of children aged 5–11 years, the VE was 68% (95% CI, 42 to 82) against COVID-19-related hospitalization in the Omicron period (median time since vaccination 34 days). For adolescents aged 12–18 years, the VE was 40% (9–60%) (median time since vaccination 162 days) [11]. In an Italian study conducted during Omicron dominance, the VE against severe COVID-19 (leading to hospitalization or death) was 41.1% (22.2–55.4%) among 5–12-year-olds [26]. Hospitalizations in the present study include all-cause hospitalizations and not only those related to COVID-19.

Vaccinated boys aged 5–11 years had a higher rate of visits to the ER within six weeks after SARS-CoV-2 infection (PERR 1.13 (1.04–1.22)), which is not easily explained by the data in the present study. This finding cannot, however, be explained by variations in health care use between the vaccinated and unvaccinated since the analyses account for health care use one year prior to vaccination.

No significant differences were found between vaccinated and unvaccinated children and adolescents in the included health outcomes, pneumonia, croup, and severe conditions (MIS-C, myocarditis, venous thromboembolism, Guillain–Barré syndrome, or encephalitis), except for an unadjusted and significant reduced risk of febrile seizures among vaccinated 5–11-year-olds. However, the mean age of the vaccinated children was higher than among the unvaccinated, which could explain the lower number of events among the vaccinated children. Previous research found that protection of the vaccine against symptomatic SARS-CoV-2 infection during the Omicron period was modest and decreased rapidly [10]. By contrast, an American study found that in adolescents aged 12–18 years, the BNT162b2 vaccine protected against critical COVID-19 with a VE of 79% (51–91%) and 20% (−25–49%) against noncritical COVID-19 during Omicron dominance [11].

There was no difference between vaccinated and unvaccinated 12–18-year-olds in any of the included outcomes in the present study. This age group was vaccinated during the spring and summer of 2021 and thus experienced a longer time between vaccination and infection than the 5–11-year-olds vaccinated from November 2021. The effectiveness of the vaccine declines with time [25]. Therefore, rather than indicating a weaker protective effect of the vaccine in adolescents than in children, this might also be explained by waning immunity from the vaccine. In conclusion, the present study found lower rates of visits to the ER and hospitalizations in vaccinated 5–11-year-old girls and boys, respectively. By contrast, 5–11-year-old vaccinated boys had a higher rate of visits to the ER. For the included health outcomes, no significant differences were found between vaccinated and unvaccinated children and adolescents, with the exception of an unadjusted and significant lower risk of febrile seizures in vaccinated 5–11-year-olds. No differences were found between vaccinated and unvaccinated 12–18-year-year-olds in any outcome. The results indicate a modest protective effect of the vaccine in terms of hospital contacts but no protective effect on health outcomes after SARS-CoV-2 Omicron infection in this population of Danish children and adolescents.

## Figures and Tables

**Table 1:** Baseline characteristics prior to a positive Omicron test.

	Age 5–11 Years ^1^		Age 12–18 Years ^1^	
	Unvaccinated	Vaccinated	Unvaccinated	Vaccinated
All n	n = 103,712	n = 64,063	n = 29,538	n = 223,325
Sex, girls (number, %)	50,708 (48.9%)	30,921 (48.3%)	14,234 (48.2%)	111,879 (50.1%)
Age vaccination date ^1^				
Age (mean, SD)	7.5 (2.0)	8.8 (1.9)	13.8 (1.7)	14.6 (1.8)
Age at Omicron infection				
Age (mean, SD)	7.6 (2.0)	9.0 (1.9)	14.1 (1.9)	15.1 (1.9)
Medical history ^2^, n/Yes %				
Asthma	5038 (4.9%)	3833 (6.0%)	1308 (4.4%)	10,017 (4.5%)
Other chronic respiratory diseases	957 (0.9%)	597 (0.9%)	49 (0.2%)	324 (0.1%)
Chronic cardio-vascular disease	393 (0.4%)	298 (0.5%)	165 (0.6%)	1329 (0.6%)
Renal diseases	354 (0.3%)	240 (0.4%)	85 (0.3%)	705 (0.3%)
Diabetes (type I or II)	177 (0.2%)	170 (0.3%)	110 (0.4%)	1139 (0.5%)
Autoimmune conditions	2492 (2.4%)	1669 (2.6%)	761 (2.6%)	5389 (2.4%)
Epilepsy	778 (0.8%)	460 (0.7%)	344 (1.2%)	2072 (0.9%)
Malignant or Immunosuppressive	422 (0.4%)	326 (0.5%)	133 (0.5%)	887 (0.4%)
Congenital diseases	2995 (2.9%)	1941 (3.0%)	459 (1.6%)	3258 (1.5%)
Admission for psychiatric condition ^3^	392 (0.4%)	280 (0.4%)	424 (1.4%)	2630 (1.2%)
Current medicine use ^4^, n/Yes %				
Inhaled short-acting beta2 agonists	2715 (2.6%)	1724 (2.7%)	512 (1.7%)	4710 (2.1%)
Inhaled corticosteroid	2105 (2.0%)	1593 (2.5%)	290 (1.0%)	3267 (1.5%)
Systemic antihistamine	1344 (1.3%)	777 (1.2%)	419 (1.4%)	4850 (2.2%)
Systemic corticosteroid	60 (0.1%)	30 (0.0%)	43 (0.1%)	409 (0.2%)
Non-Steroidal Anti-Inflammatory Drugs	525 (0.5%)	384 (0.6%)	490 (1.7%)	5747 (2.6%)
Any category of antibiotics, n/Yes (%)				
1 filled prescription	4468 (4.3%)	2178 (3.4%)	1518 (5.1%)	17,070 (7.6%)
2 filled prescriptions	866 (0.8%)	381 (0.6%)	329 (1.1%)	4345 (1.9%)
3+ filled prescriptions	265 (0.3%)	157 (0.2%)	115 (0.4%)	1770 (0.8%)

^1^ Age at possible vaccination according to the Danish age-based vaccination schedule base. Only individuals with first SARS positive test between 1 January and 31 March 2022 are included as fully vaccinated or unvaccinated prior to January 2022. Vaccination age was assigned to unvaccinated based on ages when tested SARS-2 positive and their vaccinated peers. ^2^ Information on medical history/co-morbidity was retrieved from the Danish Patient Registry and based on in-patient and out-patient diagnoses prior to the first test positive result. Both primary and secondary diagnoses are included; see Supplementary Table S2 for applied ICD-10 codes. ^3^ For psychiatric conditions only information on in-patients and primary diagnoses is included. ^4^ Current medicine use is defined as a filled prescription within 6 months prior to the date of positive test. See Supplementary Table S2 for applied ATC codes.

**Table 2:** PERR ^1^ and the number of healthcare contacts per 1000 person years (rates) among infected children with and without vaccination.

Healthcare Contact	Girls					Boys				
	Unvaccinated Rate (Number of Events)		Vaccinated Rate (Number of Events)			Unvaccinated Rate (Number of Events)		Vaccinated Rate (Number of Events)		
	Before	After	Before	After	PERR (95% CI) ^2^	Before	After	Before	After	PERR (95% CI) ^2^
**5–11 years**										
Hospitalization	0.022 (2008)	0.029 (296)	0.021 (1190)	0.026 (168)	0.96 (0.79–1.17)	0.025 (2557)	0.028 (336)	0.020 (1364)	0.018 (141)	0.79 (0.64–0.99)
Emergency department ^3^	0.120 (10,718)	0.143 (1472)	0.116 (6614)	0.127 (832)	0.92 (0.84–1.00)	0.122 (12,685)	0.131 (1566)	0.114 (7660)	0.139 (1073)	1.13 (1.04–1.22)
**12–18 years**										
Hospitalization	0.031 (1113)	0.043 (177)	0.026 (7480)	0.033 (1097)	0.92 (0.77–1.12)	0.027 (964)	0.034 (141)	0.024 (5912)	0.026 (746)	0.86 (0.71–1.02)
Emergency department ^3^	0.104 (3725)	0.124 (512)	0.095 (27,006)	0.113 (3704)	1.00 (0.92–1.11)	0.123 (4446)	0.129 (536)	0.116 (29,030)	0.128 (3667)	1.05 (0.96–1.16)

^1^ PERR (Prior event rate ratio) = ratio after (vaccinated rate after/unvaccinated rate after) divided by the ratio before (vaccinated rate before/unvaccinated rate before). ^2^ Bootstrapped 95% confidence interval, applying 200 replications. ^3^ Defined as acute visits with a duration shorter than 12 h.

**Table 3:** Results of propensity weighted (IPW) binomial regression on in-hospital severe health outcomes ^1^ within 6 weeks after infection.

Diagnosis Outcome	Vaccinated	N	Risk Ratio ^2^			
			Unadjusted ^3^ (95% CI)	*p*	IPW Weighted ^4^ (95% CI)	*p*
Severe conditions ^1^	No	133,246	1.000		1.000	
	Yes	287,374	0.31 (0.05; 1.85)	0.20	0.52 (0.08; 3.14)	0.47

^1^ Covers incident health outcomes of either MIS-C (multisystem inflammatory syndrome in children), myocarditis, venous thromboembolism, Guillain–Barré syndrome or encephalitis, combined composite binary outcome. Individuals previously diagnosed with one of these conditions were excluded (n = 18). ^2^ Risk ratio = events/N (vaccinated) divided by events/N (unvaccinated). ^3^ Results of the unadjusted binominal regression analyses with 95% confidence interval. ^4^ Results of propensity weighted binominal regression analyses, applying Inverse Treatment Probability Weighting (IPW), including age, sex, comorbidities, and current medicine use as weights.

**Table 4:** Age-group stratified results of propensity weighted (IPW) binomial regression on severe in-hospital health outcomes within 6 weeks after infection.

Diagnosis Outcome	Age-Group (Years)	Vaccinated	N	Risk Ratio ^1^					
				Unadjusted ^2^ (95% CI)		*p*	IPW Weighted ^3^ (95% CI)		*p*
Febrile Seizure ^4^	5–11	No	103,713	1.00	-		1.00	-	
	5–11	Yes	64,063	0.12 (0.04; 0.39)		<0.01	0.30 (0.09; 1.08)		0.07
	12–18	No	29,538	1.00	-		1.00	-	
	12–18	Yes	223,325	-	-	-	-	-	-
Pneumonia	5–11	No	103,713	1.00	-		1.00	-	
	5–11	Yes	64,063	0.20 (0.02; 1.62)		0.13	0.45 (0.06; 3.60)		0.45
	12–18	No	29,538	1.00	-		1.00	-	
	12–18	Yes	223,325	0.48 (0.18; 1.28)		0.14	0.44 (0.15; 1.27)		0.13
Croup	5–11	No	103,713	1.00	-		1.00	-	
	5–11	Yes	64,063	0.92 (0.51; 1.66)		0.78	1.20 (0.64; 2.26)		0.57
	12–18	No	29,538	1.00	-		1.00	-	
	12–18	Yes	223,325	0.66 (0.08; 5.66)		0.71	0.76 (0.09; 6.52)		0.80

^1^ Risk ratio = events/N (vaccinated) divided by events/N (unvaccinated). ^2^ Results of the unadjusted binominal regression analyses with 95% confidence interval. ^3^ Results of propensity weighted binominal regression analyses with 95% confidence interval, applying as weights Inverse Treatment Probability Weighting (IPW), including as weight: age, sex, comorbidities, current medicine use, along with the diagnosis outcome recorded (y/n) within the last four years prior to vaccination indexdate. ^4^ Febrile seizures occur in children aged six months up to about 5 years. Hence, any recorded febrile seizure in adolescents is considered a coding error. Thus, it has not been possible to calculate risk estimates for the adolescents 12–18 years old.

## Data Availability

Data cannot be made available for others due to privacy concerns and regulations.

## Supplementary Materials

The following supporting information can be downloaded at: https://www.mdpi.com/article/10.3390/vaccines11121766/s1, Supplementary Table S1: Health outcome measures based on hospital in- and out-patient diagnoses; Supplementary Table S2: ICD-10 and ATC codes used to define base-line characteristics: Somatic and psychiatric comorbidities, and current medicine use.
