# Supplementary material for: The Impact of Vaccination against SARS-CoV-2 on Health Outcomes and Hospital Visits after Omicron Infection in Children and Adolescents Aged 5–18 Years: A Danish Nation-Wide Cohort Study
Source: Vaccines (Basel). 2023 Nov 27;11(12):1766. doi: 10.3390/vaccines11121766 (PMC10747739; doi:10.3390/vaccines11121766)
Supplement: Supplementary file 1 [file vaccines-11-01766-s001.zip › vaccines-2711447-supplementary.pdf]

Supplementary material

Supplement to: Berg SK, Wallach-Kildemoes H, Rasmussen LR, Nygaard U, Bundgaard H, Ersbøll AK, Bering L, Thygesen LC, Nielsen SD, Christensen AV. *The impact of vaccination against SARS-CoV-2 on health outcomes and hospital visits after Omicron infection in children and adolescents aged 5-18 years: A Danish nation-wide cohort study.*

Contents supplementary

Supplementary Table S1 Health outcome measures based on hospital in- and out-patient diagnoses 3

Supplementary Table S2 ICD-10 and ATC codes used to define base-line characteristics: Somatic and psychiatric comorbidities, and current medicine use 4

Supplementary Table S1 Health outcome measures based on hospital in- and out-patient diagnoses[1]

Diagnosis category	ICD-10 diagnosis codes
MIS-C [2,3]	B972B, M303
Myocarditis[3]	I40*, I41*, I514
Venous thromboembolism [3]	I26*, I801, I802, I803, I808. I809,
Guillain-Barré syndrome [3]	G610
Encephalitis [3]	A858, A869, G04*-06*
Pneumonia[4]	A481, B012, J12*-J18*, J100
Viral/acute laryngotracheitis (croup)[4]	J050, J042, J040
Febrile seizure[4]	R560

1) Information on diagnoses corresponding to possible short-term complications from SARS-CoV-2 infection (i.e., within 6 weeks), obtained from hospital diagnoses recorded in the Danish National Patient Registry. Diagnosis-codes with Asterix include any diagnosis with the stated characters as first characters.
2) A combined outcome of MIS-C (multisystem inflammatory syndrome in children) and Kawasaki disease syndrome
3) Severe and rare outcomes combined as one binary variable, termed severe conditions.
4) Other less severe pediatric outcomes.

Supplementary Table S2 ICD-10 and ATC codes used to define base-line characteristics: Somatic and psychiatric comorbidities, and current medicine use

Comorbidities/medical history[1]	Medical history: ICD 10 codes on hospital diagnoses<br>Current medicine use: ATC codes on filled of prescriptions
Asthma	J45-J46
Other respiratory disorders	E84; J41-44; J47; J84; P27
Cardiovascular disorders	I05-I09; I20-I28; I34-I37; I42-I49; I50-I51
Renal disorders incl. dialysis	N03; N04-5; N07; N18-19; N25-N27
Diabetes mellitus I or II	E10-E11
Autoimmune conditions excl. diabetes	D510; D590; D591; D690; D693; D86; E035; E039; E050; E055; E059; E063; E065; E271; E272; E310; G04; G131; G35; G36; G61; G700; H20; I00-I02; K50; K51; K732; K743; K900; L10; L12; L130; L40; L63; L80;<br>M05-M06; M08; M30; M311; M313; M315-M317; M32-M34;<br>M350-M353; M358-M359; M45; M60
Epilepsy	G40
Malignant or Immunosuppressive	C00-C96; D70-D72; D730; D81-D84
Congenital diseases	Q00-Q07; Q20-Q28; Q30-Q34; Q60-Q64; Q90-Q99
Psychiatric conditions	F (any psychiatric primary diagnoses)
**Current medicine use[2]**	
Inhaled Short-acting Beta Agonists	R03AC02-4; R03AL01-02; R03CC02
Inhaled Corticosteroid	R03BA; R03AK; R03AL08; R03AL09
Systemic antihistamine	R06A
Systemic corticosteroid	H02AB
NSAIDs	M01 excl. M01AX
Antibiotics	J01

ICD-10 = International Classification of Diseases and Health Related Problems, 10th revision; ATC = Anatomical Therapeutical Chemical Classification; NSAIDs = non-steroidal anti-inflammatory drugs.

1) Individuals with the specified comorbidities/medical history were obtained, applying individual-level from inpatient and outpatient hospital diagnoses (primary and secondary diagnoses), recorded through ICD-10 codes in the Danish National Patient Registry prior to the positive SARS-CoV-2 test (i.e., full medical historic). Information on psychiatric conditions were restricted to primary hospital diagnoses for inpatients.
2) Individuals with current use of the specified medicines were identified from the Danish National Prescription Registry by means of recorded ATC-codes on filled prescription medicines within 6 months prior to the positive SARS-CoV-2 test.
